# Impact of Different Visual Field Testing Paradigms on Sample Size Requirements for Glaucoma Clinical Trials

**DOI:** 10.1038/s41598-018-23220-w

**Published:** 2018-03-20

**Authors:** Zhichao Wu, Felipe A. Medeiros

**Affiliations:** 1 Duke Eye Center and Department of Ophthalmology, Duke University School of Medicine, Durham, North Carolina USA; 2 Department of Ophthalmology, University of California, San Diego, La Jolla, California, USA; 3 Centre for Eye Research Australia, Royal Victorian Eye and Ear Hospital, East Melbourne, Australia; 4 Ophthalmology, Department of Surgery, The University of Melbourne, Melbourne, Australia

## Abstract

Visual field testing is an important endpoint in glaucoma clinical trials, and the testing paradigm used can have a significant impact on the sample size requirements. To investigate this, this study included 353 eyes of 247 glaucoma patients seen over a 3-year period to extract real-world visual field rates of change and variability estimates to provide sample size estimates from computer simulations. The clinical trial scenario assumed that a new treatment was added to one of two groups that were both under routine clinical care, with various treatment effects examined. Three different visual field testing paradigms were evaluated: a) evenly spaced testing, b) United Kingdom Glaucoma Treatment Study (UKGTS) follow-up scheme, which adds clustered tests at the beginning and end of follow-up in addition to evenly spaced testing, and c) clustered testing paradigm, with clusters of tests at the beginning and end of the trial period and two intermediary visits. The sample size requirements were reduced by 17–19% and 39–40% using the UKGTS and clustered testing paradigms, respectively, when compared to the evenly spaced approach. These findings highlight how the clustered testing paradigm can substantially reduce sample size requirements and improve the feasibility of future glaucoma clinical trials.

## Introduction

The ability to accurately detect disease progression is paramount in preventing functional disability in eyes with glaucoma, and visual field testing remains the most important tool for this task. However, the inherent variability of this test^1–4^ continues to pose a significant challenge for the timely detection of progressive visual field loss^5^. Detection of progression requires the separation of true change from measurement variability. However, as glaucoma is generally a relatively slowly progressive disease, true changes can be largely confounded by variability, when assessed over a relatively short period of time. This not only hinders individual patient management, but can also be troublesome in the setting of clinical trials seeking to use visual function endpoints as an outcome measure. In order to detect a sufficient number of endpoints, it may be necessary to employ large sample sizes or conduct very frequent tests over relatively long periods of time. This can be burdensome to patients and may reduce the feasibility of such trials.

Even though conventional studies have generally employed a testing paradigm requiring evenly spaced testing visits over time, a recent study by Crabb & Garway-Heath^6^ proposed that clustering visual field tests at the beginning and end of a follow-up (described as a “wait-and-see” approach) could improve detection of progression and thus reduce sample size requirements for clinical trials using visual field endpoints^6^. However, clustering of visual field tests at only two time points, such as at the beginning and end of the observation period, may bring unwarranted consequences, such as the delayed detection of visual field progression due to the lack of interim visits. In addition, any participant that becomes lost to follow-up before completing the last set of visual fields with this testing paradigm cannot contribute to the study endpoints.

Nonetheless, the helpful notion of clustering visual field tests at the beginning and end of the trial period was incorporated into the design of the United Kingdom Glaucoma Treatment Study (UKGTS)^7^. The UKGTS was a randomized clinical trial designed to evaluate the beneficial effect of latanoprost versus placebo in delaying visual field progression. The testing paradigm of the study consisted of clusters of visual fields at baseline, as well as 18 and 24-months. However, the study also included one regular visual field test at each follow-up visit occurring at approximately 2–3 month intervals^7^. Whilst this paradigm is likely to be an improvement from an evenly spaced approach, the extent to which it would reduce sample size requirements is unknown. Also, as the UKGTS still included burdensome intermediary visits occurring every couple of months, it remains to be determined if using a testing paradigm that includes fewer of those intermediary visits, but which would employ clustering of tests in each one could be beneficial in terms of further reducing sample size requirements and decreasing the number of visits.

The purpose of this study was to evaluate sample size requirements for clinical trials using visual field endpoints using different testing paradigms. We demonstrate that a hybrid paradigm modeled after the “wait-and-see” approach, but modified to include few intermediary visits, is a powerful design for detecting endpoint differences when investigating the effect of new therapies compared to existing standard of care.

## Results

### Participant Characteristics

**Figure 1:**
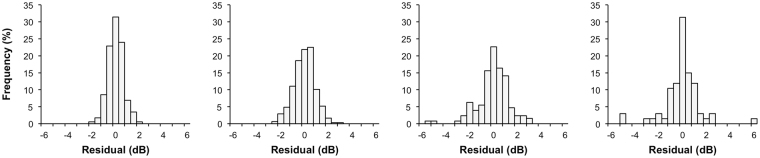
Distribution of the residuals (representing estimates of visual field variability) at four estimated visual field mean deviation (MD) bins.

A total of 353 eyes from 247 glaucoma participants were included in this study to obtain estimates of rates of change in visual field mean deviation (MD) in a clinically treated population as well as estimates of test-retest visual field variability. The participants were seen over 7.9 ± 2.4 visits (range, 6 to 16 visits) during a 3-year follow-up period. At baseline, they were on average 68.0 ± 10.6 years old (range, 33 to 92 years old), and the median (interquartile range [IQR]) of MD and PSD for these eyes was −3.43 dB (−7.74 to −1.36 dB) and 3.75 dB (2.32 to 8.65 dB) respectively. The median MD rate of change of the eyes used in the simulations was −0.22 dB/year (IQR = −0.68 to 0.17 dB/year), and distribution of the residuals at four representative MD bins is shown in Fig. 1.

### Sample Size Requirements of Different Visual Field Clustering Paradigms

Table 1 shows required sample sizes to identify treatment effects ranging from 20% to 50% for the 3 different visual field testing paradigms. Compared to the conventional evenly spaced testing paradigm, the sample size needed to detect a statistically significant treatment effect was reduced by approximately 17–19% using the UKGTS testing paradigm, but up to 39–40% with the clustered testing paradigm. For instance, a total of 391, 323 and 237 participants would be needed per group to have 90% power to detect a 30% treatment effect in reducing the rate of change when using the evenly spaced, UKGTS and clustered testing paradigms, respectively.

**Table 1:** Sample size required to detect a statistically significant treatment effect using different testing paradigms.

New Treatment Effect	Sample Size Required Per Group		
	Evenly Spaced Paradigm	UKGTS Paradigm	Clustered Paradigm
20%	890	726	532
30%	391	323	237
40%	235	193	142
50%	152	123	91

UKGTS=United Kingdom Glaucoma Treatment Study.

### Detection of Visual Field Progression at the Individual Level

**Figure 2:**
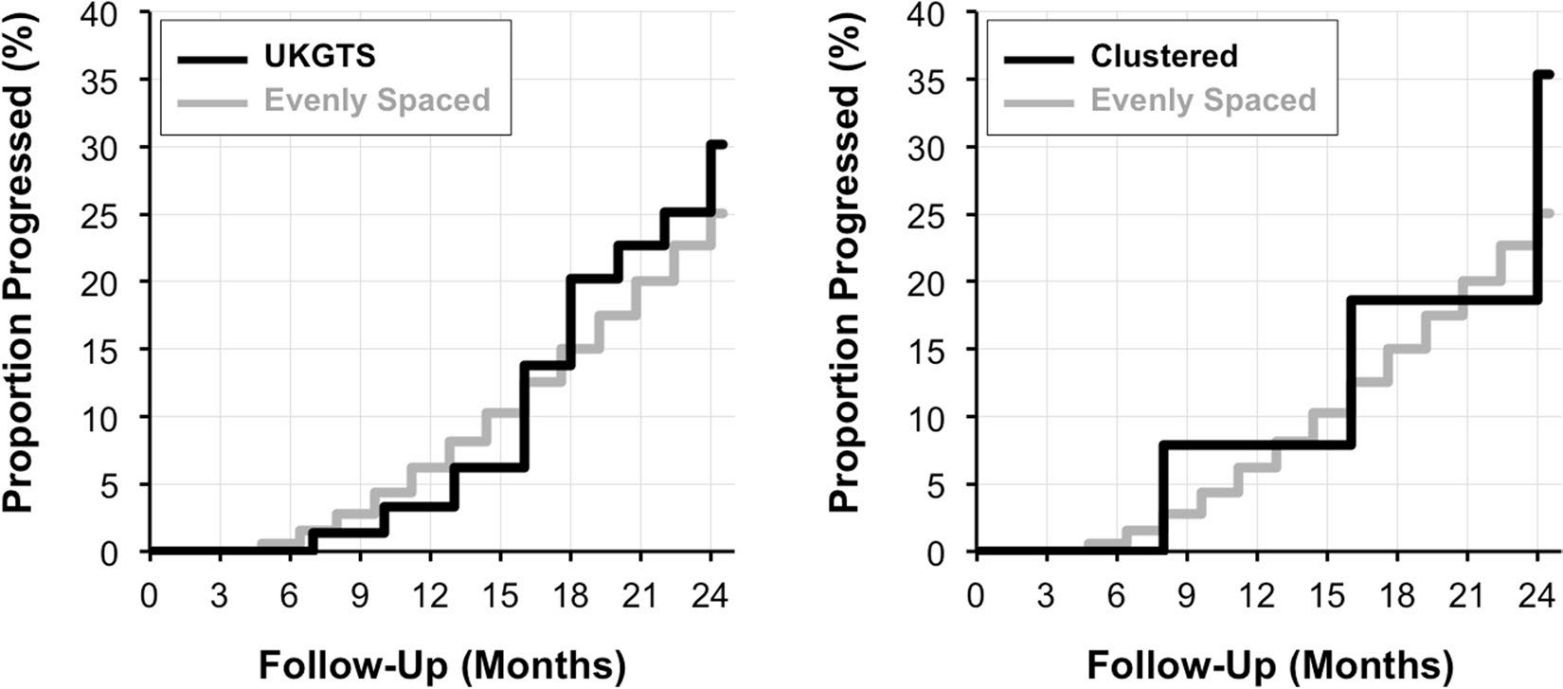
Cumulative proportion of eyes detected as having progressed using the United Kingdom Glaucoma Treatment Study (UKGTS) and clustered testing paradigms compared to the evenly spaced paradigm over time.

Individuals exhibiting visual field progression typically require clinical management changes, and thus become excluded from the remainder of a trial. It is thus crucial that each testing paradigm can detect visual field progression in a timely manner. The cumulative percentage of eyes identified as having progressed was determined for each testing paradigm and illustrated in Fig. 2. By the end of the follow-up period, both the UKGTS and clustered testing paradigms detected a greater cumulative number of eyes progressing (30% and 35%, respectively) than the evenly spaced paradigm (25%). More importantly, the cumulative rate of progression was also generally higher during each follow-up visit prior to the end of the trial period for the clustered paradigm than at the same time point with the evenly spaced paradigm.

## Discussion

This study demonstrated that the sample size requirements for a short-term clinical trial were reduced using the UKGTS testing paradigm when compared to a conventional evenly spaced paradigm (by approximately 18%), but even further reduced by the clustered testing paradigm described in this study (by approximately 40%) when between-group trend-based analysis was used as the outcome measure. The reduction in sample size requirements with the clustered paradigm was achieved without compromising its capacity for early detection of visual field progression at the individual level during the follow-up. This clustered testing paradigm therefore provides another method for further improving the feasibility of glaucoma clinical trials using visual field endpoints, including those that seek to evaluate the effect of new neuroprotective treatments^8–11^.

The reduction in sample size requirements with the clustered paradigm described in this study can be attributed to the well-established statistical concept that slope estimates are optimally achieved when the observations are equally allocated at each end of the experimental region, minimizing the variance of the slope estimate^12,13^. These findings are in agreement with the previous study by Crabb & Garway-Heath^6^. However, clustering of tests only at the baseline and final visits with the previous “wait-and-see” approach could potentially be harmful to some patients included in a trial as there would be only one opportunity to detect progression, at the end of the trial. In fact, when the recent UKGTS incorporated the notion of clustering tests in its follow-up scheme, it ensured that intermediary visits with regular testing were still included^7^. However, if a clustered paradigm could be made such as to preserve the power to detect individual progression over time and reduce sample size requirements with the same number of tests, it could potentially reduce trial costs. In fact, our proposed clustered visual field testing paradigm that required only two intermediary visits detected approximately 40% more eyes as having progressed than the evenly spaced paradigm at matched specificities by the end of a simulated 2-year clinical trial period, whilst reducing sample size requirements by approximately 40% as well. Even by reducing the number of tests at the beginning and end of the trial period from six to four tests to improve its feasibility, the sample size requirements were still lower (being 237 and 304 required per group, respectively,  for a 30% treatment effect, for instance), highlighting how cost savings can also be achieved by reducing the total number of visits required.

Note that the clustered testing paradigm performed in this study assumes that visual field tests for each cluster would be performed on separate days within a short timeframe, rather than performing multiple tests on the same day, as we used longitudinal variability estimates in our simulations rather than intrasession estimates. In practical terms, this may look like testing participants several times over a few weeks at the beginning and end of the trial period. Whilst this testing paradigm may appear burdensome, the current evenly spaced testing approach that spreads the same number of tests into testing every 1.5 to 2 months can be considered burdensome as well. Further studies are needed to better understand the patient perspective on this issue, but a compelling reason to favor the clustered testing paradigm from a patient’s perspective would be the increased ability to detect visual field progression at the individual level.

Furthermore, note also that our findings should not be interpreted as suggesting that the UKGTS protocol was suboptimal, especially given that the study design was modified after the trial had begun, to extend the trial period from 18 months to 24 months (due to the slower rate of recruitment than initially expected)^7^. As such, the testing paradigm used would likely have been modified and optimized had it been originally designed for a 24-month trial period. Instead, the findings of our study show that a testing paradigm used in a historical clinical trial that includes some level of clustering of visual field tests performs better than an evenly spaced approach, and that it could be improved even further for future trials using the proposed clustered paradigm .

Previous clinical trials in glaucoma have commonly used event-based analysis methods, such as the Guided Progression Analysis, to establish visual field endpoints. Event-based methods provide a binary categorization of whether progression has occurred during the follow-up of a particular eye or individual. The cumulative proportion of eyes showing progression (i.e. reaching the endpoint) with a new drug is then compared to that of the control arm, usually by survival analysis methods. Unfortunately, such approach is very inefficient and results in very large sample size requirements. In our study, we assumed that treatment effects would be evaluated by comparing the overall rate of change between the treatment and control groups, by a trend-based analysis of visual field MD over time using linear mixed effects modeling; similar procedures have been used extensively in clinical trials in other areas of medicine^10,14–17^. In a recent study, we demonstrated that this trend-based approach would reduce sample size requirements in glaucoma trials by approximately 85–90% compared to event-based approaches^18^.

In this study, we also evaluated treatment efficacy by comparing the rate of visual field progression between two groups assumed to be under routine, conventional glaucoma treatment. Such conventional treatment would, by itself, lower the rate of progression when compared to patients without treatment or under placebo. Therefore, demonstrating a treatment effect in this scenario is substantially more challenging than when comparing a new drug with placebo. That is, it is harder to demonstrate differences when the rates of change in the two groups are already relatively slow to begin with. This is an important consideration when evaluating the reported sample sizes from our analyses. However, it should be emphasized that such comparison would be more applicable for future clinical trials in view of potential ethical concerns that would be raised from proposed placebo-controlled trials in patients with glaucoma.

**Figure 3:**
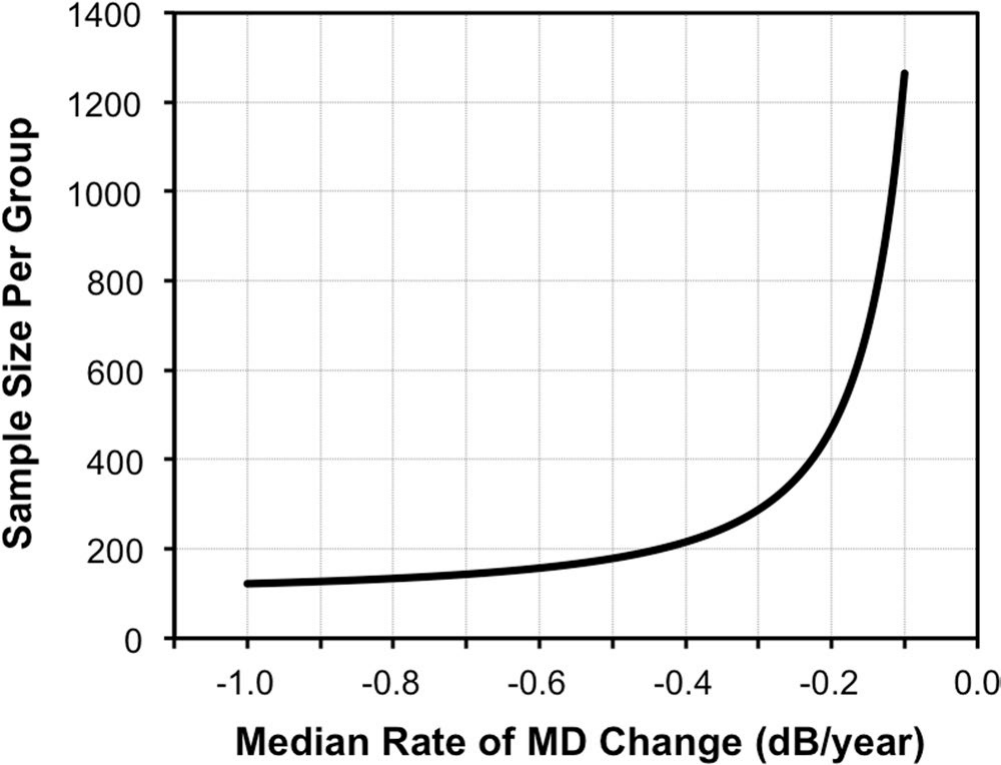
The sample size required to detect a 30% new treatment effect with 90% power for various median rates of visual field mean deviation (MD) change of the simulation cohort, using an evenly spaced testing paradigm.

Another important point to consider when using the sample size estimates provided by this study is to note that the actual estimates will vary depending on the characteristics of the participants included and number of tests performed. For instance, we previously reported that 277 participants would be required per group to detect 30% new treatment effect with 90% power using the between-group trend-based analysis, when a total of 10 tests were performed over a 2-year period (with two tests at baseline and subsequent testing at 3-month intervals)^18^. However, this study found that 394 participants are required per group to also detect a 30% new treatment effect using the evenly spaced testing paradigm, although 16 tests were performed over a 2-year period. This discrepancy is most likely attributed to the difference in the visual field progression rates of the participants included in the two studies (with the median MD rate of change being −0.22 and −0.57 dB/year in this study and our previous study^18^ respectively), with sample size requirements being smaller in a cohort with a faster rate of progression^9^. We therefore obtained sample size estimates assuming different median rates of change in a population, illustrating in Fig. 3 the established non-linear relationship between sample size requirements and median rate of MD change.

Note also that the sample size estimates were obtained in this study using longitudinal variability estimates obtained from participants tested at a more conventional clinical follow-up interval (approximately every 6-months) than the testing frequencies evaluated in this study. It is thus possible that participants included in a clinical trial with a higher frequency of testing may become more experienced over time, and thus exhibit a lower extent of measurement variability. As such, the power to detect a significant treatment effect may actually be higher when using the sample size estimates obtained using the longitudinal cohort.

Nonetheless, the findings in this study builds upon the work in our previous study^18^, demonstrating that the feasibility of future glaucoma trials using visual field endpoints can be further improved by using a clustered testing paradigm to better estimate the difference in the rate of visual field loss between groups. Furthermore, a recent review^19^ has also argued that evaluating the rate of visual field change as an outcome measure is clinically meaningful, on the basis of an accumulating body of evidence that has shown that the rate of visual field loss due to glaucoma – independent of the magnitude of visual field loss itself – is associated with functional disability^20–26^. The findings of this study can be generalized to other clinical trial designs based on their relevant needs, through understanding the principles of the clustered testing paradigm. Specifically, sample size requirements are minimized when tests are equally allocated at each end of the observation period with this approach. Therefore, even though we have included only two tests during two intermediary visits in this study (on the basis that such a design would be suitable for evaluating the perimetric glaucoma eyes included in this study), the specific testing protocol can be modified on the basis of different needs. For instance, the intermediary visits could be modified to have three tests (rather than two) in order to improve the ability to detect progression at the individual level during these visits, at the expense of having one less test at each end of the observation period (which would increase the required sample size). Alternatively, a clinical trial may prefer to have a longer trial duration but maintain the same number of intermediary visits (and thus a longer follow-up interval) if participants at low-risk of progression (e.g. glaucoma suspects) were evaluated, or reduce the number of intermediary visits and allocate those tests to each end of the trial period to further reduce the sample size requirements.

In summary, this study demonstrates that a clustered visual field testing paradigm that includes follow-up visits during the observation period reduces sample size requirements by approximately 40% compared to a paradigm where the same number of tests are performed at regular intervals. This sample size reduction was achieved without compromising on the safety factor of detecting visual field progression early during the observation period, and can thus substantially improve the feasibility of future glaucoma clinical trials.

## Methods

### Participants

Participants included in this study were evaluated as part of a longitudinal study that was designed to investigate structural and functional damage in glaucoma. Institutional review board approval by the University of California, San Diego was obtained for this study, and it adhered with the Declaration of Helsinki and Health Insurance Portability and Accountability Act. Written informed consent was obtained from all the participants in this study following an explanation of all the test procedures.

At each visit, all participants underwent a comprehensive ophthalmologic examination including a medical history review, visual acuity measurements, visual field testing, slit-lamp biomicroscopy, ophthalmoscopic examination, intraocular pressure measurements, gonioscopy and stereoscopic optic disc photography. All participants were required to have open angles on gonioscopy, a best-corrected visual acuity of 20/40 or better, and be 18 years of age or older. Subjects were excluded if they presented any other ocular or systemic disease that could affect the optic nerve or the visual field.

This study included only glaucoma eyes with confirmed visual field damage at baseline and corresponding glaucomatous optic nerve damage as assessed by masked grading of the optic nerve appearance on stereophotographs using methods described previously^27^. Eyes were considered to have visual field damage if they had ≥ 3 consecutive abnormal visual field tests (defined as having a pattern standard deviation value at *P* < 0.05, or glaucoma hemifield test being outside normal limits)^28^, and only tests including and subsequent to the first of the three consecutive abnormal visual field tests were included in this study.

### Visual Field Testing

Visual field tests were performed using the 24–2 strategy with the Swedish Interactive Thresholding Algorithm on the Humphrey Field Analyzer II-i (Carl Zeiss Meditec, Inc., Dublin, CA, USA). Visual fields were evaluated for artifacts including eyelid or rim artifacts, inattention, fatigue or learning effects, improper fixation, or for any evidence that another disease other than glaucoma (e.g. homonymous hemianopia) was affecting the results; any tests with such artifacts were excluded from this study. Visual fields were considered unreliable and also excluded if > 33% fixation losses or false negative errors (with the exception for false negative errors when visual field MD was less than −12 dB), or > 15% false positive errors were recorded for a test. This study included only eyes with ≥ 6 eligible tests within a 3-year follow-up period to ensure that a sufficient number of tests were included for estimating the rate of visual field change^29^ within a similar timeframe as evaluated in this study.

### Data Analysis

This study used visual field data from the longitudinal cohort to extract frequencies and magnitudes of rates of visual field change in glaucomatous eyes followed over time under standard clinical care. In addition, the cohort was also used to extract information about visual field variability that could be used to simulate the progression scenarios necessary for sample size calculations. Sample size calculations were estimated by comparing the rate of visual field MD change over time between the two groups in a clinical trial using linear mixed models (LMMs), including random intercepts and random slopes to account for individual deviations when estimating a population-average change. We have recently demonstrated that such approach is much more powerful and results in vast reductions in sample size requirements for clinical trials as compared to event-based approaches such as Guided Progression Analysis (GPA; Carl Zeiss Meditec, Inc., Dublin, CA, USA)^18^.

**Figure 4:**
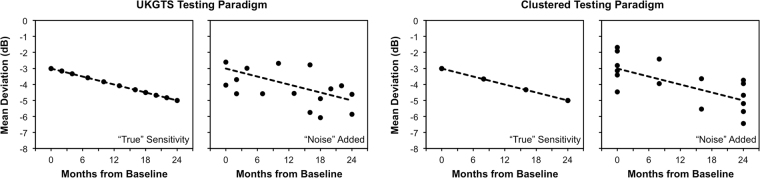
Illustrations of the method used to reconstruct “real-world” visual field mean deviation (MD) values using the United Kingdom Glaucoma Treatment Study (UKGTS; left) and clustered (right) testing paradigms. In each example, the “true” sensitivity at each time point was calculated using an estimated MD slope and intercept, before “noise” (or measurement variability) was added to each of these values.

To determine the sample size and power required for detecting a significant treatment effect under different scenarios, a computer simulation model was developed to reconstruct “real-world” visual field outcomes; this approach has been described recently^5^. In brief, the first step of developing this model was to estimate the range of rates of visual field MD change over time (or “signal”) by performing ordinary least squares (OLS) linear regressions on MD values over time for all the individual eyes in this study. Visual field variability (or “noise”) was then estimated by the residuals from the OLS model, which were obtained by subtracting the observed values from the OLS fitted values. The variability estimates were then grouped into 1-dB bins according to the fitted values. To reconstruct the “real-world” visual field MD at each time point, the “true” visual field MD at each time point was determined by the estimated MD slope and intercept for each eye. The “noise” component (or residuals) was then randomly selected based on the “true” MD rounded to the nearest 1-dB and added to the “true” MD value, and thus simulating “real-world” visual field test results; illustrations of this procedure are shown in Fig. 4. Simulations were performed only for eyes that had an estimated baseline MD of ≥ −15 dB in order to exclude eyes that had advanced glaucomatous visual field damage.

### Clinical Trial Testing Paradigms

Visual field results were then reconstructed for a scenario where a clinical trial was conducted over a 2-year period, and 16 visual field tests were performed over this period (as per the UKGTS study design^7^). Three different testing paradigms were investigated in this study that included the same number of tests (to enable fair comparisons between them):

1. Evenly Spaced Paradigm: for 16 tests to be evenly spaced over a 24-month period, testing was performed every 1.6 months, which would correspond to approximately every one and a half months in practice.
2. UKGTS Paradigm: testing was clustered at the beginning and end of the clinical trial period, with two tests performed at the 0 and 2 month visits, and at the 16, 18 and 24 month visits. One test was otherwise performed at the 4, 7, 10, 13, 20 and 22 month visits.
3. Clustered Paradigm: this paradigm is similar to the “wait-and-see” approach proposed in a previous study^6^ where testing is clustered at the tail-ends of the trial, but includes testing at 8-month intervals to provide an opportunity for follow-up at regular intervals; this follow-up interval is consistent with clinical recommendations for glaucoma patients under treatment^30^. As such, 6 tests were performed at baseline and 24-months, in addition to two tests every 8-months during the follow-up.

**Figure 5:**
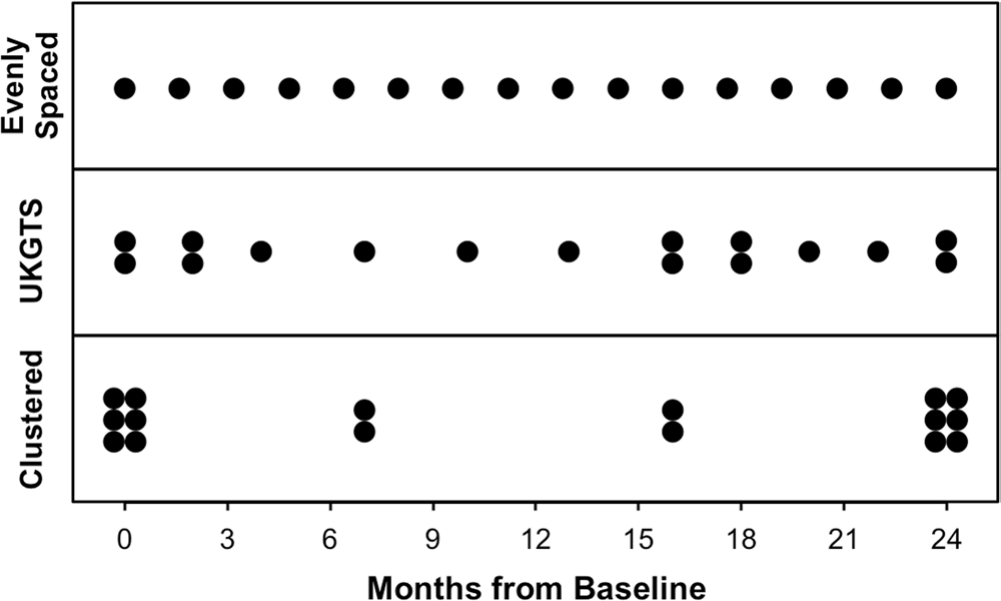
Illustration of the three designs compared in the study: evenly spaced, United Kingdom Glaucoma Treatment Study (UKGTS) and clustered testing paradigms.

The follow-up schemes of the three testing paradigms are illustrated in Fig. 5.

### Detecting Visual Field Progression in Individual Eyes

To determine whether each testing paradigm can allow visual field progression to be detected throughout the duration of the trial period, progression was evaluated by performing OLS linear regression analyses for each simulated eye at the different time points throughout the study. To ensure equivalency between the different testing paradigms, the specificities for the criterion used to define progression were matched. This was performed by generating 100 sequences of visual field results for each eye in the simulation cohort, assuming that the true MD slope of change was 0 dB/year for all eyes (i.e. that the eyes were in fact stable). We then obtained cut-offs for *P*-values for the OLS regression that ensured matched specificity. This also provided an opportunity to investigate the ability of each clinical testing paradigm to sensitively detect visual field progression over time, to ensure that the clustered paradigm would not significantly delay the detection of such progressive changes.

### Simulated Treatment Effects and Sample Size Estimates

This study evaluated the sample size requirements for clinical trials using the 3 different paradigms described above, for a trial scenario where a new treatment was added to one of the two groups that were both under routine treatment with currently available therapies. Note that this approach requires larger sample sizes than the simple comparison of a new treatment versus placebo, due to the effect of routine treatment in reducing rates of progression. However, it is a more realistic scenario in view of the ethical concerns of using a placebo arm in future clinical trials in glaucoma. We investigated sample sizes necessary to detect treatment effects resulting in 20%, 30%, 40% or 50% reduction in mean rates of change with the new treatment.

The required sample size estimates were obtained by simulating 1000 sequences of clinical trials for each testing paradigm, which included between 10 to 1000 participants per group. During each simulated trial, eyes with different baseline and rate of MD change were randomly selected without replacement from the cohort of eligible eyes, and the same eyes were used in the two treatment arms, to match the participant characteristics of the two treatment groups (as performed in randomized clinical trials). For each simulated trial, the beneficial effect of the new treatment was evaluated using LMMs to determine the difference in rate of MD change over time between the treatment groups. The statistical significance of the new treatment effect was determined by examining the interaction between the treatment variable and time (in other words, determining whether there was a significant difference in the rate of MD change between the two groups), and a significance level of 5% for a two-tailed test was used. The estimate of the required sample size for each design was obtained by observing the sample size where a statistically significant beneficial treatment effect could be detected in 90% of the simulated sequences (or with 90% power) was determined.

### Data Availability Statement

The datasets generated during and/or analysed during the current study are available from the corresponding author on reasonable request.

## References

[1] Heijl A, Lindgren A, Lindgren G (1989). Test-retest variability in glaucomatous visual fields. Am. J. Ophthalmol..

[2] Chauhan BC, Johnson CA (1999). Test-retest variability of frequency-doubling perimetry and conventional perimetry in glaucoma patients and normal subjects. Invest. Ophthalmol. Vis. Sci..

[3] Wall M, Woodward KR, Doyle CK, Artes PH (2009). Repeatability of automated perimetry: a comparison between standard automated perimetry with stimulus size III and V, matrix, and motion perimetry. Invest. Ophthalmol. Vis. Sci..

[4] Russell RA, Crabb DP, Malik R, Garway-Heath DF (2012). The relationship between variability and sensitivity in large-scale longitudinal visual field data. Invest. Ophthalmol. Vis. Sci..

[5] Wu Z, Saunders LJ, Daga FB, Diniz-Filho A, MEdeiros FA (2017). Frequency of Testing to Detect Visual Field Progression Derived Using a Longitudinal Cohort of Glaucoma Patients. Ophthalmology.

[6] Crabb DP, Garway-Heath DF (2012). Intervals between visual field tests when monitoring the glaucomatous patient: wait-and-see approach. Invest. Ophthalmol. Vis. Sci..

[7] Garway-Heath DF (2013). The United Kingdom Glaucoma Treatment Study: a multicenter, randomized, placebo-controlled clinical trial: design and methodology. Ophthalmology.

[8] Chang EE, Goldberg JL (2012). Glaucoma 2.0: neuroprotection, neuroregeneration, neuroenhancement. Ophthalmology.

[9] Quigley HA (2012). Clinical trials for glaucoma neuroprotection are not impossible. Curr. Opin. Ophthalmol..

[10] Sena, D. F. & Lindsley, K. Neuroprotection for treatment of glaucoma in adults. *Cochrane Database Syst*. *Rev*. **2** (2013). 10.1002/14651858.CD006539.pub3 PMC4261923 23450569

[11] Levin LA, Crowe ME, Quigley HA (2017). Neuroprotection for glaucoma: Requirements for clinical translation. Exp. Eye Res..

[12] Daniel C, Heerema N (1950). Design of experiments for most precise slope estimation or linear extrapolation. Journal of the American Statistical Association.

[13] Gaylor D, Sweeny H (1965). Design for optimal prediction in simple linear regression. Journal of the American Statistical Association.

[14] Kastelein JJ (2007). Effect of torcetrapib on carotid atherosclerosis in familial hypercholesterolemia. N. Engl. J. Med..

[15] Schrier RW (2014). Blood Pressure in Early Autosomal Dominant Polycystic Kidney Disease. N. Engl. J. Med..

[16] Chataway J (2014). Effect of high-dose simvastatin on brain atrophy and disability in secondary progressive multiple sclerosis (MS-STAT): a randomised, placebo-controlled, phase 2 trial. Lancet.

[17] Hodis HN (2016). Vascular Effects of Early versus Late Postmenopausal Treatment with Estradiol. N. Engl. J. Med..

[18] Wu, Z. *et al*. Improving the Feasibility of Glaucoma Clinical Trials With Trend-Based Analysis of Visual Field Change Between Groups as an Endpoint. *Invest*. *Ophthalmol*. *Vis*. *Sci*. ARVO E-Abstract: 2465 (2017).

[19] De Moraes CG, Liebmann JM, Levin LA (2016). Detection and measurement of clinically meaningful visual field progression in clinical trials for glaucoma. Prog. Retin. Eye Res..

[20] Lisboa R (2013). Association between rates of binocular visual field loss and vision-related quality of life in patients with glaucoma. JAMA Ophthalmol.

[21] Gracitelli, C. P. B. *et al*. Association Between Progressive Retinal Nerve Fiber Layer Loss and Longitudinal Change in Quality of Life in Glaucoma. *JAMA Ophthalmol* (2014). 10.1001/jamaophthalmol.2014.5319 PMC4533995 25569808

[22] Medeiros FA (2015). Longitudinal changes in quality of life and rates of progressive visual field loss in glaucoma patients. Ophthalmology.

[23] Abe RY (2016). The Impact of Location of Progressive Visual Field Loss on Longitudinal Changes in Quality of Life of Patients with Glaucoma. Ophthalmology.

[24] Abe RY (2015). Frequency Doubling Technology Perimetry and Changes in Quality of Life of Glaucoma Patients: A Longitudinal Study. Am. J. Ophthalmol..

[25] Diniz-Filho A (2016). Fast Visual Field Progression Is Associated with Depressive Symptoms in Patients with Glaucoma. Ophthalmology.

[26] Baig S (2016). Association of fast visual field loss with risk of falling in patients with glaucoma. JAMA Ophthalmol.

[27] Sample PA (2009). The African Descent and Glaucoma Evaluation Study (ADAGES): design and baseline data. Arch. Ophthalmol..

[28] Kuang TM, Zhang C, Zangwill LM, Weinreb RN, Medeiros FA (2015). Estimating Lead Time Gained by Optical Coherence Tomography in Detecting Glaucoma before Development of Visual Field Defects. Ophthalmology.

[29] Gardiner SK (2013). Series Length Used during Trend Analysis Affects Sensitivity to Changes in Progression Rate in the Ocular Hypertension Treatment Study. Invest. Ophthalmol. Vis. Sci..

[30] Prum BE (2016). Primary Open-Angle Glaucoma Preferred Practice Pattern® Guidelines. Ophthalmology.

